# Differential lipids in pregnant women with subclinical hypothyroidism and their correlation to the pregnancy outcomes

**DOI:** 10.1038/s41598-021-99252-6

**Published:** 2021-10-04

**Authors:** Jingjing Li, Yajuan Xu, Zongzong Sun, Yanjun Cai, Biao Wang, Miao Zhang, Yanjie Ban, Xiaofeng Hou, Yingqi Hao, Qian Ouyang, Bo Wu, Mengqi Wang, Wentao Wang

**Affiliations:** Department of Obstetrics and Gynecology, The Third Affiliated Hospital of Zhengzhou University, Zhengzhou, Henan China

**Keywords:** Immunology, Biomarkers, Diseases, Endocrinology, Medical research

## Abstract

Subclinical hypothyroidism (SCH) has become a prevalent complication in pregnancy. Recent research links SCH to disturbed thyroid lipid profile; however, it is unclear how lipid metabolism disorders contribute to the pathogenesis of SCH during pregnancy. Thus, we used nontargeted lipidomics to identify and compare the lipids and metabolites expressed by pregnant women with SCH and healthy pregnant women. Multivariate analysis revealed 143 lipid molecules differentially expressed between the SCH group and the control group. Based on fold change, 30 differentially expressed lipid metabolites are potential biomarkers. KEGG pathway enrichment analysis showed that the differentially expressed metabolites participate in several pathways, including response to pathogenic Escherichia coli infection, regulation of lipolysis in adipocytes, metabolic pathways, glycerophospholipid metabolism, and fat digestion and absorption pathways. Correlation analyses revealed sphingomyelin (SM) and phosphatidylcholine (PC) positively correlate to tumor necrosis factor-α (TNF-α), C-reactive protein (CRP), and interleukin-6 (IL-6), while phosphatidylglycerol (PG), and phosphatidylinositol (PI) negatively correlate with them. In addition, PG positively correlates to birth weight. Thus, the lipid profile of pregnant women with SCH is significantly different from that of healthy pregnant women. Lipid molecules associated with the differential lipid metabolism, such as SM, phosphatidylethanolamine (PE), and PI, should be further investigated for their roles in the pathogenesis of SCH in pregnancy, as they might be targets for reducing the incidence of adverse pregnancy outcomes.

## Introduction

Subclinical hypothyroidism (SCH) in pregnancy refers to the elevation of serum thyroid-stimulating hormone (TSH) in pregnant women with normal levels of free thyroxine (FT4) and total T4. Patients with SCH usually do not have obvious clinical symptoms or physical signs, so the diagnosis of SCH depends mainly on laboratory examinations. The prevalence of SCH during pregnancy has been increasing year by year. Its adverse effects on pregnancy outcomes are becoming apparent. They include spontaneous abortion^1^, preeclampsia^2^, placental abruption, premature rupture of membranes, low birth weight^3^, fetal distress, preterm birth^4^, and abnormal neuropsychological and intellectual development^5^. The pathogenesis of SCH remains unclear, despite being associated with nitric oxide (NO)^6^, Nesfatin-1^7^, gene silencing mediated by microRNAs^8^, inactivation of the thyroid-stimulating hormone receptor gene^9^, and the gut–thyroid axis^10^. In summary, SCH during pregnancy has a high incidence, lacks definite symptoms and physical signs, and causes obvious adverse pregnancy outcomes. However, research on its pathogenesis is still lacking.

Although previous serum and urine metabolomic studies provide some clues about the pathogenesis of SCH at the metabolic level, the pathogenesis of SCH during pregnancy is still unclear, especially in terms of lipid metabolism. Zhang et al. (2018)^11^ fed a high-fat diet (HFD) or chow diet to male Sprague–Dawley rats for 18 weeks. Examination of their thyroid function showed that HFD rats had hypothyroidism, and the liquid chromatography (LC)–mass spectrometry (MS) analysis showed that HFD rats had a disturbed thyroid lipid profile. Lee et al. (2015) and Shao et al. (2014)^12,13^ also pointed out that excessive nutrition might be a pathogenic factor of hypothyroidism, which suggests the thyroid gland is another victim of lipotoxicity. The association between SCH and lipids has become more apparent in recent years. What is unclear is how lipid metabolism disorders contribute to the pathogenesis of SCH during pregnancy. Therefore, the main purpose of this study was to use LC–MS to analyze the characteristics of the lipid profile of pregnant women with SCH to investigate SCH pathogenesis and provide hints of targets that might reduce the incidence of adverse pregnancy outcomes.

## Materials and methods

### Study subjects

Pregnant women who received regular perinatal health care in the outpatient department of the Third Affiliated Hospital of Zhengzhou University and gave birth in the hospital between July 2019 and January 2020 were randomly selected. They included 30 pregnant women with SCH (SCH group) in late pregnancy and 30 healthy pregnant women (control group) in late pregnancy who met the inclusion criteria.

### Inclusion criteria

**(**1) The thyroid function levels of the SCH group met the diagnostic criteria in the 2017 guidelines of the American Thyroid Association for the diagnosis and management of thyroid disease during pregnancy and the postpartum^14^ and the reference-range criteria developed by the Department of Clinical Laboratory of the Third Affiliated Hospital of Zhengzhou University (11.5 < FT4 < 22.7 pmol/L, TSH > 4.0 mIU/L). The control group included pregnant women who had normal thyroid function and did not have other obstetric complications. (2) All pregnant women were in the late pregnancy period.

### Exclusion criteria

The exclusion criteria were as follows: (1) Patients who had incomplete data. (2) Patients who had central hypothyroidism. (3) Patients who had other obstetric complications. (4) Patients who were taking antithyroid drugs or thyroid hormone replacement. (5) Patients who had severe systemic diseases or were recovering from thyroiditis. (6) Patients who, in the last 3 months, used thyroid function affecting drugs, including metoclopramide, iodine supplements, domperidone, amiodarone, and lithium carbonate tablets. (7) Patients who had a medical history of liver diseases, malignant tumors, diabetes mellitus, and hereditary hyperlipidemia. (8) Patients who took lipid-lowering drugs in the last 3 months, such as fibrates, statins, or Xuezhikang capsules. (9) Patients who had had intestinal surgery. (10) Patients who were aged < 18 years. (11) Patients who tested positive for the thyroid peroxidase antibody.

#### Note

Central hypothyroidism refers to hypothyroidism caused by reduced production and secretion of thyrotropin-releasing hormone or TSH resulting from a hypothalamus or pituitary gland disorder.

##### Statement

(1) All enrolled subjects volunteered and signed informed consent forms. This study was approved by the Ethics Committee of the Third Affiliated Hospital of Zhengzhou University. (2) All methods were carried out in accordance with relevant guidelines and regulations.

### Specimen collection

All pregnant women fasted for 8–12 h before blood collection. A total of 5 mL blood from the median cubital vein was collected in ethylenediaminetetraacetic acid–coated tubes. After collection, the collection tube was gently inverted four times, wrapped in aluminum foil, and temporarily stored in a 4 °C refrigerator. Blood samples were centrifuged within 2 h of sample collection in a low-temperature centrifuge at 4 °C and 1600 × *g* for 10 min. After centrifugation, the supernatant (plasma) was aliquoted into several centrifuge tubes and stored in a − 80 °C freezer. Later, we sent samples to the BGI Group for lipidomic examination.

### Data collection

The basic data of the two groups of pregnant women, including body mass index (BMI) before pregnancy, BMI at enrollment, gravidity, parity, delivery mode, neonatal sex, birth weight (BW), and Apgar scores at 1 and 5 min, were collected. In addition, clinical data were collected during sample collection, including serum TSH, fasting blood glucose (FBG), CRP, IL-6, and TNF-α.

### Major instruments and reagents

Instruments used in this study included an ultrahigh-performance liquid chromatograph (UPLC) (Waters 2D UPLC, Waters, USA), a high-resolution mass spectrometer (Q Exactive, Thermo Fisher Scientific, USA), chromatographic column: ACQUITY UPLC CSH C18 (1.7 μm, 2.1*100 mm, Waters, USA), a low-temperature ultracentrifuge (Centrifuge 5430, Eppendorf), a vortex (QL-901, Qilin Beier instrument, China), a water purifier (Milli-Q Integral, Millipore Corporation, USA), and a refrigerated vacuum concentrator (Maxi Vacbeta, GENE COMPANY). Reagents used in this study included LC–MS-grade (Thermo Fisher Scientific, USA) methanol (A454-4), acetonitrile (A996-4), isopropanol (A461-4), ammonium formate (17843-250G, Honeywell Fluka, USA), and formic acid (50,144–50 ml, DIMKA, USA) and water purified by a water purifier.

### Extraction of lipid molecules

After samples were slowly thawed at 4 °C, 100 μL of each sample was pipetted into a 96-well plate. Next, 300 μl isopropanol (precooled to − 20 °C) and 10 μl SPLASH Lipidomix internal standard were added and vortexed to homogeneity for 1 min. Samples were kept at − 20 °C overnight and then centrifuged at 4 °C and 4000 rpm for 20 min. The supernatant was collected and placed in a sample tube. Next, 10 μL supernatant was collected from each sample and merged to form the quality control (QC) sample.

### UPLC-MS analysis

#### UPLC materials and methods

The CSH C18 column (1.7 μm 2.1*100 mm, Waters, USA) was used for chromatography. The mobile phases of the positive ion mode were a water solution (solution A) containing 10 mM ammonium formate, 0.1% formic acid, and 60% acetonitrile and a solution (solution B) containing 10 mM ammonium formate, 0.1% formic acid, 90% isopropanol, and 10% acetonitrile. The mobile phases of the negative ion mode were a water solution (solution A) containing 10 mM ammonium formate and 60% acetonitrile and a solution (solution B) containing 10 mM ammonium formate, 90% isopropanol, and 10% acetonitrile. The following gradient was used for elution: 0–2 min, 40–43% solution B; 2 ~ 2.1 min, 43% ~ 50% solution B; 2.1 ~ 7 min, 50% ~ 54% solution B; 7 ~ 7.1 min, 54% ~ 70% solution B; 7.1 ~ 13 min, 70% ~ 99% solution B; 13 ~ 13.1 min, 99% ~ 40% solution B, 13.1 ~ 15 min, 40% solution B. The flow rate was 0.35 mL/min, the column temperature was 55 °C, and the sample load was 5 μL.

#### MS materials and methods

The Q Exactive mass spectrometer (Thermo Fisher Scientific, USA) was used for primary and secondary MS data acquisition. The range of the mass-to-charge ratio in MS scanning was 200–2000, the primary resolution was 70,000, the automatic gain control was 3e6, and the maximum injection time was 100 ms. According to the precursor ion intensity, the top 3 was selected for fragmentation. The secondary MS data were collected. The secondary resolution was 17,500, the automatic gain control was 1e5, the maximum injection time was 50 ms, and the stepped normalized collisional energy was set as 15, 30, and 45 eV. The settings of electrospray ionization were a sheath gas flow rate of 40, an aux gas flow rate of 10, a spray voltage (|kV|) of 3.80 in the positive ion mode and 3.20 in the negative ion mode, a capillary temperature of 320 °C, and an aux gas heater temperature of 350 °C.

To provide more reliable experimental results during instrument detection, samples were randomly sorted to minimize systemic errors. One QC sample was inserted for every 10 samples.

### Statistical methods

Raw data generated by the LC–MS/MS detection were input into LipidSearch v.4.1 (Thermo Fisher Scientific, USA) for MS data analysis. The data matrix contained the lipid molecule identification results and quantitative results. Identification and peak extraction of individual samples were first performed using LipidSearch v.4.1, and then peak alignment was performed on all samples. The raw data obtained from LipidSearch were input into metaX for data pretreatment and subsequent analyses. SPSS 25.0 software was used to process basic clinical data of pregnant women in two groups. Normally distributed quantitative data are described as mean ± standard deviation (χ ± s), and differences between groups were compared using the independent-samples t-test. Nonnormally distributed quantitative data are expressed as median (M) and quartile (Q), and differences between groups were compared using the Mann–Whitney U-test. Categorical variables are described as frequency, and the differences between groups were determined by the chi-squared test. Correlations were calculated by Spearman analysis.

## Results

### General clinical data and pregnancy outcomes

This study enrolled 30 patients in the SCH group and 30 patients at the same stage of pregnancy in the control group. Table 1 provides the general clinical data and pregnancy outcomes of subjects in the two groups. The differences in age, BMI before pregnancy, BMI at enrollment, gravidity, parity, delivery mode, and fasting blood glucose between the SCH and control groups were not significant. For the pregnancy outcomes of subjects in the two groups, the differences in neonatal sex and Apgar scores at 1 and 5 min were also not significant. However, birth weight (BW) was significant (*p* = 0.036).

**Table 1:** Comparison of general clinical data and pregnancy outcomes between the SCH group and the control group.

General characteristics	SCH Group(n = 30)	Control(n = 30)	*p* value
Maternal age, year*	30.57 ± 4.21	29.80 ± 3.43	0.443
BMI before pregnancy, Kg/m^2^*	21.62 ± 2.92	21.69 ± 2.85	0.921
BMI at enrollment, Kg/m^2^*	25.95 ± 5.72	26.95 ± 3.38	0.410
Gravidity, n*	2.17 ± 1.39	1.97 ± 1.35	0.574
Parity, n*	0.40 ± 0.50	0.37 ± 0.56	0.808
**Delivery mode**			0.793
Normal delivery, n	13	12	
Cesarean delivery, n	17	18	
FBG, mmol/L*	4.59 ± 0.59	4.55 ± 0.51	0.792
**Neonatal sex**			0.795
Female, n	13	14	
Male, n	17	16	
Birth weight, g*	3213.53 ± 289.10	3374.80 ± 294.35	0.036
**Apgar scores**			
1 min	9.47 ± 1.85	9.70 ± 0.65	0.518
5 min	9.60 ± 1.83	9.87 ± 0.35	0.436

*Data are expressed as means ± standard deviation. *p* < 0.05 was considered statistically significant. Apgar scores: It is the scoring method made according to the child's physical condition after birth, composed of five clinical components: Activity, Pulse, Grimace, Appearance, and Respiration. Each component receives 0, 1, or 2 points based on predetermined criteria, with a maximum score of 10.

### Difference analysis for the SCH group and the control group

**Figure 1:**
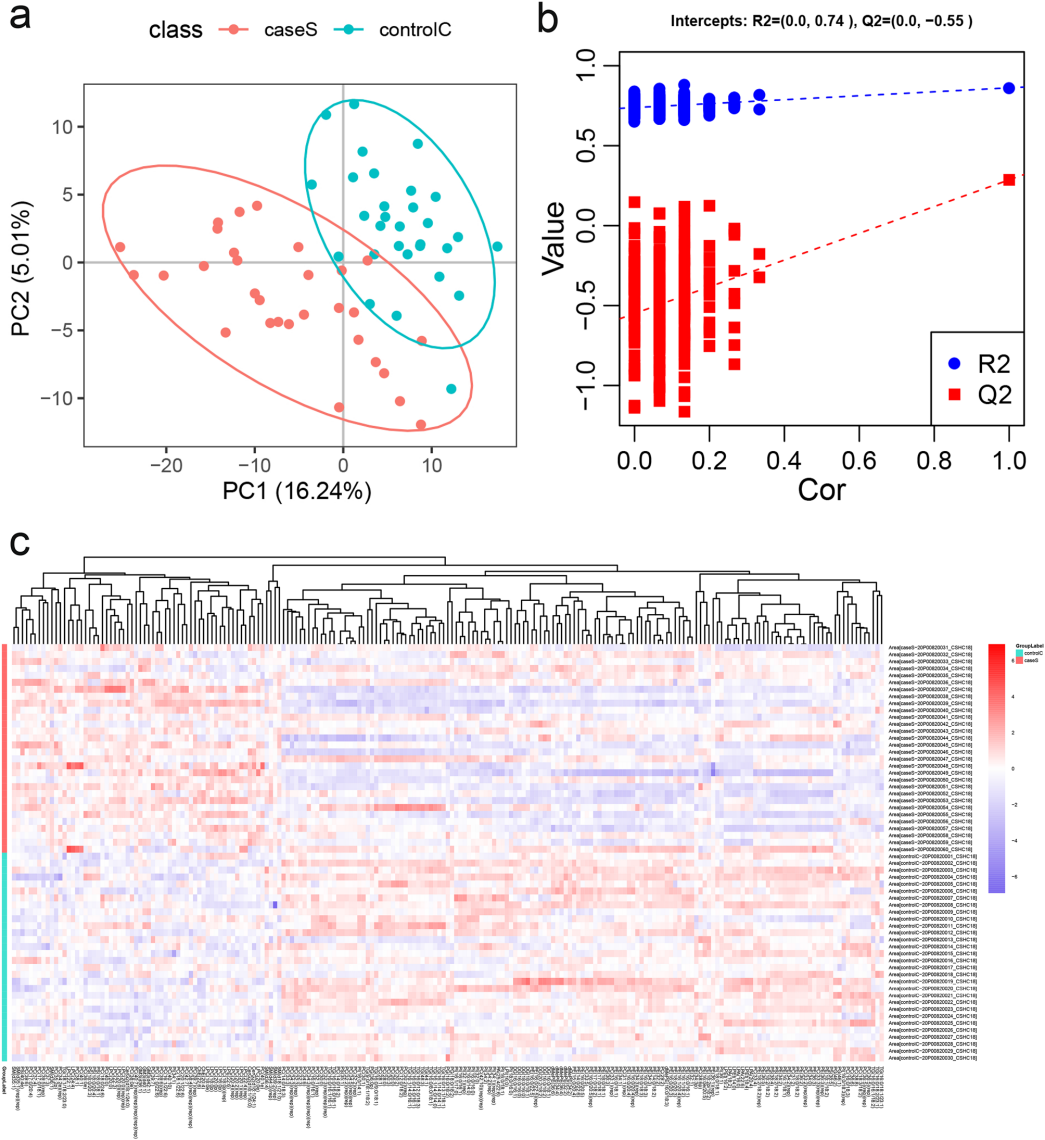
Difference analysis for the SCH group and the control group. (**a**) The PLS-DA model score plot; (**b**) response sequencing test plot of the PLS-DA analysis model. (**c**) The cluster analysis diagram shows the aggregation trend in lipid profile between the SCH and control groups. (Note: One point in (**a**) corresponds to one sample, green represents the control group, and red represents the SCH group. Each column in (**c**) represents a differential ion, and each row represents a sample. The different shades of color indicate the intensities, of which the blue indicates low-intensity and red high-intensity).

To validate the significant differences between the SCH group and the control group, the supervised partial least squares–discriminant analysis (PLS-DA) multivariate method was used to remodel and analyze the two groups of data (Fig. 1a). The results showed that most members of the two groups could be separated (R2Y-0.862 and Q2 = 0.286). In model validation (Fig. 1b), R2 was (0.0, 0.74) and Q2 was (0.0, − 0.55). The validation results showed that this model was stable and reliable. Differential lipid molecules were subjected to cluster analyses (Fig. 1c). The results of hierarchical clustering analysis for the SCH and control groups (represented in the green and red color respectively) showed a sort of clustering in some areas. This phenomenon indicated that the distribution of lipid metabolites was different among the two groups. Furthermore, the heat map also presented variations in the relative concentrations of various lipid metabolites. This information provided an overview of lipid metabolites differences between the SCH and control groups. However, a more detailed analysis is needed to better understand the lipidomics associated with SCH.

### Screening of differential lipid molecules

**Figure 2:**
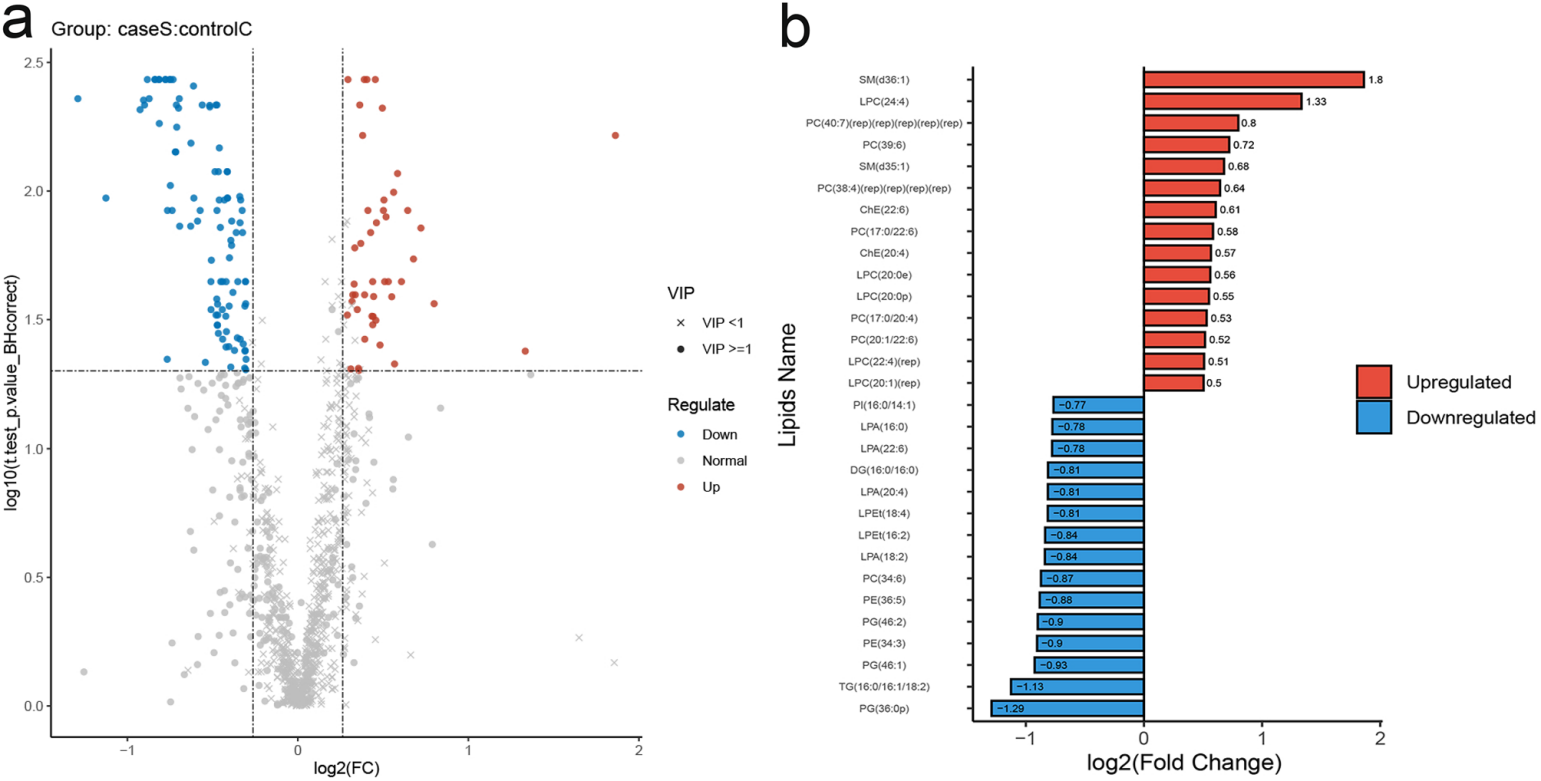
Screening of differential metabolites. (**a**) Volcano plot shows the number of dysregulated lipids in the SCH group compared to the control group. (**b**) Potential biomarkers. Note: (rep) indicates we repeated the identification results of this lipid.

The screening conditions of the differential lipid molecules were as follows: (1) The VIP of the first two principal components in the PLS-DA model ≥ 1, (2) FC ≥ 1.2 or ≤ 0.83, and (3) *t*.test_*p*.value_BHcorrect < 0.05. The volcano plot used log_2_(FC) as the X-axis and -log_10_(*t*.test_*p*.value_BHcorrect) as the Y-axis (Fig. 2a). It showed that compared with the healthy group, a total of 143 differential metabolites were identified in the SCH group, including 46 upregulated metabolites and 97 downregulated metabolites.

Based on FC and biological significance, 30 compounds (the 15 most upregulated and 15 most downregulated) were shortlisted as potential biomarkers (Fig. 2b). The upregulated compounds included SM (d36:1), lysophosphatidylcholine (LPC) (24:4), PC (40:7)(rep) × 5, PC (39:6), SM (d35:1), PC (38:4)(rep) × 4, cholesteryl ester (ChE) (22:6), PC (17:0/22:6), ChE (20:4), LPC (20:0e), LPC (20:0p), PC (17:0/20:4), PC (20:1/22:6), LPC (22:4)(rep), LPC (20:1)(rep). The downregulated compounds included PG (36:0p), TG (16:0/16:1/18:2), PG (46:1), PE (34:3), PG (46:2), PE (36:5), PC (34:6), lysophosphatidic acid (LPA) (18:2), lyso-phosphatidylethanol (LPEt) (16:2), LPEt (18:4), LPA (20:4), diglycerides (DG) (16:0/16:0), LPA (22:6), LPA (16:0), PI (16:0/14:1).

### Pathways analyses and correlation between potential biomarkers and clinical data

**Figure 3:**
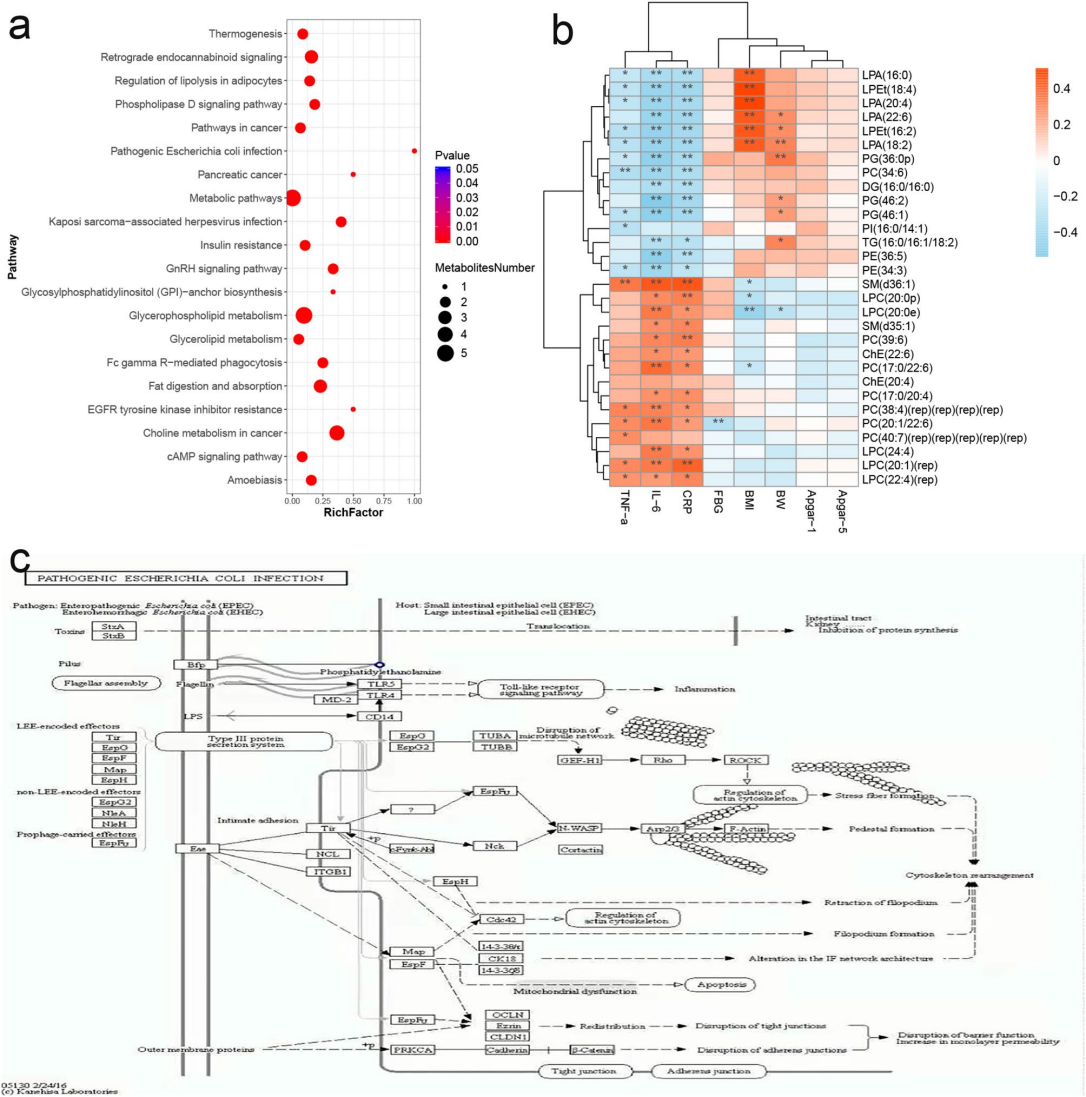
Pathways analyses and correlation between potential biomarkers and clinical data. (**a**) Scatter plot of KEGG enrichment. (**b**) Correlation between potential biomarkers and clinical data. (**c**) Pathogenic Escherichia coli infection response pathway. (Note: *BMI* BMI at enrollment; *BW* birth weight; Apgar-1: Apgar scores at 1 min; Apgar-5: Apgar scores at 5 min. *, *p* < 0.05; **, *p* < 0.01).

We used the Kyoto Encyclopedia of Genes and Genomes (KEGG) database to link the differential metabolites to metabolic pathways. The results indicated that differential metabolites in the SCH group and the control group participated in many pathways (Fig. 3a). Some of the main ones were the response to pathogenic *Escherichia coli* infection, regulation of lipolysis in adipocytes, metabolic pathways, glycerophospholipid metabolism, and fat digestion and absorption pathways.

We performed Spearman correlation analyses on the potential biomarkers with clinical data (Fig. 3b). LPA and LPEt had a positive correlation with BMI at enrollment, while LPC had a negative correlation. SM, PC, and LPC showed a positive correlation with TNF-α, CRP, and IL-6, while PG, LPEt, LPA, PI, and different subclasses of PC showed a negative correlation with them. PG, LPA and TG had a positive correlation with BW, while LPC (20:0e) had a negative correlation. Moreover, there was no significant correlation between 30 differential metabolites and Apgar scores at 1 min and 5 min.

## Discussion

Lipids are essential metabolites for living organisms and have many critical cellular functions. They can reflect the metabolic status of cells. Lipid metabolism in pregnant women changes significantly to meet the fetus's needs of growth and development and ensure the health of the mother. Dysregulated lipid metabolism causes vascular endothelial damage, energy supply impairment^15^, and imbalance of immune and thyroid functions. In this study, we performed a nontargeted plasma lipidomic analysis on pregnant women with and without SCH to investigate the pathogenesis of SCH during pregnancy.

The association between the thyroid gland and the lipidome is not well understood. Guidetti et al. (2006)^16^ extracted total lipids from the mitochondria of rats with hypothyroidism and showed that the proportion of PC increased and the proportion of PE decreased. The LC–MS study of Liu et al. (2020)^17^ showed that PC, SM, and PE levels in patients with hypothyroidism increased. Our PLS-DA plot showed that the lipidomes of pregnant women with SCH and healthy pregnant women were different. The volcano plot and the bar graph showed that the levels of PE, SM, and PC (40:7)(rep) × 5, PC (39:6), PC (38:4)(rep) × 4, PC (17:0/22:6), PC (17:0/20:4), PC (20:1/22:6) were higher in the SCH group than in the control group. The results are consistent with previous studies. But our study also found the levels of PC (34:6) were lower in the SCH group. Although they both belong to phosphatidylcholine, they are different lipid molecules, so they may play different roles. Some studies^18,19^ have indicated that TSH and TG are positively correlated. Screening of differential metabolites between the two groups in our study showed that the levels of TG (16:0/16:1/18:2) and DG (16:0/16:0) decreased in the SCH group. The reasons for this discrepancy might be the small sample size and differences in experimental techniques. In addition, the TG (16:0/16:1/18:2) and DG (16:0/16:0) obtained in this study were subclasses of TG and DG and did not represent the serum levels of total TG and DG. Therefore, our results indicate that the lipidomes of the SCH group and the control group were significantly different, and their lipid metabolism could distinguish these two groups.

PE affects many cellular processes, including cellular stability and the functions of many membrane proteins, and is a critical regulatory factor of cell membrane fluidity. Our KEGG pathway analysis in Fig. 3c showed that the pathogenic Escherichia coli infection response pathway is significant, and this may be associated with the higher PE levels in our SCH group. The reason being, in response to pathogenic Escherichia coli infection, during the contact process between bacterial flagellin and Toll-like receptor (TLR) 4 and TLR5 in gut epithelial cells, PE is an essential stimulus of the Toll-like receptor signaling pathway to induce inflammation further. Our previous studies^20^ have shown that gut flora had significant differences between pregnant women with hypothyroidism and healthy pregnant women. The relative abundances of gut Proteobacteria, Gammaproteobacteria, and Provotella in women with hypothyroidism during pregnancy were all higher than those in the control group at the same stage. In recent years, gut microbiota composition and function changes have been considered a hallmark of metabolic damage^21^. The extremely diverse metabolites gut microbiota produced, such as lipopolysaccharides and endotoxins, could reduce the integrity of cell connections^22^. They enter the systemic circulation through the leaky gut mechanism to induce T cell activation, resulting in a series of immune and inflammatory responses^23,24^. In addition, studies^25,26^ have indicated that inflammation and the TLR pathway are associated with thyroid functions.

SM levels are mainly altered by endoplasmic reticulum (ER) stress, and SM plays important physiological roles in cell growth, differentiation, senescence, and signal transduction^27^. ER stress^28^ might be a very important mechanism during induction of SCH by a HFD, and the IRE1α/XBP-1 pathway is involved. In this study, we observed that SM in the SCH group was higher than that in the control group and positively correlated with IL-6. Five different sphingomyelinases can hydrolyze SM to exert various biological functions. Sphingomyelinases are influenced by TNF-α^29^ in vivo. Furthermore, the PI level in pregnant women with SCH during pregnancy was lower than that in healthy pregnant women. In thyroid cells, PI participates in intracellular TSH signal transduction through phosphatidylinositol (3,4,5)-trisphosphate (PIP-3)^30^. Lipid kinases in the phosphoinositide 3-kinase (PI3K) family can promote TH1 lymphocytes to produce higher levels of interferon-γ and TNF-α secretion to stimulate release of chemokine ligand (CXCL) 10 from thyroid cells to initiate and maintain the autoimmune process in the body^31–33^. In our correlation analysis, PI and TNF-α showed a negative correlation. SCH during pregnancy is an immune-metabolic disease. Zhu et al. (2019)^34^ showed that IL-6 and TNF-α levels in SCH patients were significantly higher than those in healthy individuals. Therefore, metabolic disorders of SM and PI in pregnant women might cause SCH during pregnancy by affecting the autoimmune process.

In conclusion, we summarize a possible pathogenic mechanism of SCH during pregnancy. (1) During the contact process between bacterial flagellin and TLR4 and TLR5 on gut epithelial cells, PE is an important stimulator of the TLR signaling pathway leading to inflammation and further influencing the development of the disease. (2) ER stress^28^ might be a very important mechanism during the induction of SCH by HFD. In addition, the IRE1α/XBP-1 pathway is involved, and SM plays an important role. (3) PI participates in intracellular TSH signal transduction through the PIP-3 signaling pathway^30^. Disruption of PIP-3 signal transduction further influences immune processes to affect thyroid functions.

Some studies have shown that hypothyroidism during pregnancy could increase the risk of premature delivery, low birth weight infants, and fetal distress^3,4^. Labarre et al. (2020)^35^ found that maternal blood lipid level during pregnancy can affect the umbilical cord blood lipid group and its relationship with body weight. Our study found that PG, LPA and TG were positively correlated with birth weight, while LPC (20:0e) was negatively correlated, and there was a significant difference in birth weight between the SCH group and the control group. Therefore, we can speculate that PG and other differential lipid metabolism molecules can affect the newborn's birth weight via umbilical cord blood. The results of our study did not find any difference between the two groups of neonatal Apgar scores at 1 min and 5 min, and there was no correlation between the potential biomarkers and Apgar scores at 1 min and 5 min. Therefore, this study did not find a significant impact of gestational hypothyroidism on fetal distress.

The strengths of this study are: (1) we ensured drugs that influence the lipid profile were not a factor in this study, and (2) we tested the association between the serum lipid profiles and clinical characteristics in pregnant women at the smallest possible interval. But, of course, this study also had limitations:

The sample size was small.

This study had regional specificity due to factors such as dietary habits and geographical differences.

Specimen collection took place after discovering the thyroid function disorders, so we could not determine the causal relationship between the lipid metabolism disorder and thyroid dysfunction.

This study only included samples in late pregnancy before the subjects took medication. Therefore, we could not study the dynamic changes to serum lipid metabolism in the whole pregnancy and after drug treatment. These considerations also provide direction for our future studies.

In short, we analyzed the serum lipidomes of two groups of pregnant women and found significant differences in the lipid profiles of pregnant women with SCH and healthy pregnant women. Lipid metabolism disorder in pregnant women might result in the development of SCH during pregnancy by affecting inflammation and immune responses through an ER stress mechanism, PIP-3 signaling pathway disruption, and gut flora metabolism. Therefore, lipid molecules associated with the differential lipid metabolism, such as SM, PE, and PI, should be further investigated for their role in the pathogenesis of SCH in pregnancy, as they might be targets for reducing the incidence of adverse pregnancy outcomes.
